# Farm Animal Welfare on the Mind and Meat in the Diet: Who Are These Consumers?

**DOI:** 10.3390/ani16132110

**Published:** 2026-07-07

**Authors:** Mariske P. Hajer, Jeroen Borghuis, Job M. Molendijk, Joost Meekes, Antoon Opperhuizen

**Affiliations:** 1 Chair of Risk Assessment and Risk Communication, Maastricht University, 6229 ER Maastricht, The Netherlands; antoon.opperhuizen@maastrichtuniversity.nl; 2 Office for Risk Assessment & Research, Netherlands Food and Consumer Product Safety Authority, 3511 EW Utrecht, The Netherlands; j.borghuis@nvwa.nl (J.B.); j.m.molendijk@nvwa.nl (J.M.M.); j.h.a.meekes@nvwa.nl (J.M.)

**Keywords:** farm animal welfare concern, diet, meat consumption, background factors, associations

## Abstract

We investigated the association between farm animal welfare (FAW) concern and self-classified meat consumption behavior and consumers’ socio-demographic, animal-contact-related, and ideological background factors. Covariate analyses and a machine-learning algorithm, Random Forest, were used to analyze complex, non-linear relationships between the data from 2116 Dutch households. FAW concern often turns out to be moderate to strong but is not simply associated with reduced meat consumption. Political orientation showed the strongest association with FAW concern and diet. Left-wing orientation is positively associated with FAW concern and less frequent or no meat consumption. Christian affiliation does the opposite, while non-Christian religious affiliation is weakly associated with vegetarian or vegan diets. Men more likely report an omnivorous diet and less FAW concern, while women report a vegetarian or vegan diet and higher concern. Voting left or center, Christian affiliation, and higher education are linked to a flexitarian diet. Lower education is associated with an omnivorous diet. In both rural and urban regions, income and household composition have little to no associations with both dietary self-classification and FAW concern. Respondents with regular animal contact show relatively strong FAW concern but no association with self-identified diet. Our results suggest that reducing or abstaining from meat is most strongly associated with ideological orientations. FAW concern alone is not expressed in diet.

## Simple Summary

Public concern about farm animal welfare (FAW) is high, yet more than 9 out of 10 Dutch people eat meat from farm animals. Although concern about the welfare of farm animals is widespread, it is not directly associated with reduced meat consumption. We analyzed consumers’ background factors, self-reported meat-eating behavior, and FAW concern using a survey of more than 2000 Dutch households. Consumers reported how often they eat meat and their level of FAW. We correlated their answers with background factors such as age, education, income, household size, number of children, voting behavior, and animal contact. Political orientation has the strongest association with both FAW concern and self-reported meat consumption frequency. Voters for left-wing parties show greater FAW concern and eat meat less often. Women express higher FAW concern than men and eat meat less frequently. Christian religious affiliation is associated with lower FAW concern and higher meat consumption, while atheists and people with other religious views show the opposite. Despite high FAW concern, in the Netherlands, eating meat, whether as omnivores or as flexitarians, is still a normal thing to do for people with various backgrounds.

## 1. Introduction

Results from the European Union’s (EU) Special Eurobarometer “Attitudes of Europeans towards animal welfare” showed that 84% of citizens across Europe believe farmed animal welfare (FAW) should be better protected [1,2]. Academic research also reports a high, and growing, public concern about FAW [3,4]. The public debate has continued since the Brambell report in 1965 about the Enquiry into the Welfare of Animals kept under Intensive Livestock Husbandry Systems (https://archive.org/details/b3217276x, accessed on 28 May 2026). In many Western countries, this led to laws and regulations to reduce risks to farm animal welfare [5,6,7]. Farm animal welfare (FAW) refers to the physical and mental state of animals kept for food production in relation to the conditions in which they live and die, consistent with the definition of animal welfare provided by the World Organisation for Animal Health (WOAH) [8]. In the present study, FAW concern refers to the degree of concern individuals express about the welfare of farm animals. Beyond its relevance for farm animal welfare itself, FAW concern may provide insight into the role of animals in contemporary society. The extent to which individuals include farm animals within their moral circle may be reflected in the degree of concern they express about their welfare. As such, FAW concern may be associated with food consumption patterns and societal debates about livestock production and food systems. Increased public concern about FAW did not reduce meat consumption in societies in Europe or worldwide, nor did it increase the percentage of vegans and vegetarians. In fact, average meat consumption has grown over recent decades, both in total and per capita in many countries [7,9,10]. This process, sometimes called “meatification”, has increased the number of farmed animals worldwide. Previous research indicates that FAW concern of individual consumers does not necessarily translate into reduced meat consumption [11,12,13,14,15,16,17]. This apparent inconsistency, caring for animals while still eating them, has been called the meat paradox [18,19]. Eating meat seems to be rooted in cultural, social, and personal context and is still perceived as normal at the expense of the welfare of farm animals [18,20,21]. Consumers may experience dissonance, as they value animal welfare while continuing to consume meat, but may justify this behavior by perceiving meat consumption as natural, normal, necessary, and nice [22]. When asked, consumers underscored the importance of high animal welfare standards, but acknowledged that they did not consider this when buying meat [13]. The willingness to pay more for meat from animals raised under higher FAW standards was shown to be mainly motivated by the perceived positive effects of animal-friendly production methods on meat quality and health, rather than by FAW concerns [23,24]. These consumers perceive this meat as healthier, safer, tastier, more hygienic, more authentic, more traditional, and more environmentally friendly. However, concerns about the treatment of farm animals, together with pro-environmental beliefs, may lead to less positive attitudes towards meat consumption [25,26]. An online survey across five European countries showed that consumers consider FAW an important purchasing factor, alongside other product-related considerations such as food safety, environmental sustainability, and price [27]. A wide range of considerations is involved in meat-related decision-making, including hedonic factors such as taste, habit, health aspects, ethical considerations such as animal welfare and ecological sustainability, and social and contextual influences such as norms, habits, income, and life stage [28,29,30,31,32,33,34,35,36,37,38]. Ethical concerns, including FAW, may motivate consumers to reduce meat consumption, but this relationship is complex and not always consistent. FAW concern is unlikely to be the sole motivation for initiating dietary changes [15,16].

In high-income countries such as the Netherlands, people who do not consume meat can be classified as “vegetarians or vegans of choice” [39]. In the European Union, about 4.6% of the population identified as vegetarian or vegan in 2019, and 4% in 2023 [40] (https://www.beuc.eu/sites/default/files/publications/beuc-x-2020-042_consumers_and_the_transition_to_sustainable_food.pdf (accessed on 28 May 2026), https://www.beuc.eu/reports/farm-animal-welfare-what-consumers-want (accessed on 28 May 2026), 2020 and 2024 [41,42]). Other studies report comparable figures [17,43]. About 2% or fewer of Europeans are self-reported pescatarians [43]. Instead of becoming vegetarian or vegan, some consumers reduce their meat consumption frequency, which is often called a flexitarian diet [44,45,46]. Flexitarians form a broad category, and definitions and eating patterns differ across studies and populations [47,48,49,50]. Between 2011 and 2019, in the Netherlands, self-declared flexitarians rose from 13% to nearly 43% [51]. However, the share of flexitarians eating meat at most twice a week at dinner dropped from 15.5% to 9.7%. Thus, in practice, self-identified flexitarians may eat amounts of meat like regular omnivores. Because there are no clear definitions of flexitarianism, comparing percentages across countries and studies is difficult [52].

Research directly linking FAW concern to meat consumption within the same population sample remains limited. Several national surveys assessed both FAW concern and meat consumption [53,54] (https://www.beuc.eu/reports/farm-animal-welfare-what-consumers-want (accessed on 28 May 2026) [42]). These surveys found that people with higher FAW concern tend to eat less meat. However, these associations are not always strong and may be affected by social desirability bias [13].

In 2014, Loughnan et al. published “The Psychology of Eating Animals” [19], where they examined the characteristics of the eaters (people), the eaten (animals), and the eating behavior. Variation in FAW concern and meat consumption has been linked to several individual background characteristics. In general, three main categories emerge from various overviews: 1. socio-demographic factors, such as age, gender education, income, household composition, and urbanization; 2. factors related to interaction with animals, including pet ownership or farming experience; 3. ideological background, like political orientation and religious affiliation. Table 1 provides a narrative overview of background factors previously associated with FAW concern and meat consumption. Relevant studies were identified through literature searches using combinations of keywords related to farm animal welfare, animal welfare concern, meat consumption, vegetarianism, veganism, and dietary behavior. Additional publications were identified through backward and forward citation tracking (snowball sampling). The overview is intended to provide context for the present study and does not represent a systematic review of the literature.

Findings regarding background characteristics are not always consistent across studies. Such inconsistencies may reflect differences in national and cultural contexts, study populations, sample sizes, measurement approaches, and the operationalization of both dietary behavior and FAW-related constructs.

Background factors such as age, gender, and income do not ensure group uniformity; substantial variation exists within groups [55]. These factors often interact and jointly shape FAW concern and meat consumption, rather than acting separately [56].

**Table 1:** Narrative overview of background factors associated with FAW concern and meat consumption in previous studies.

Factor Category	Factor	FAW Concern (Refs)	Meat Consumption (Refs)
Socio-demographic	Gender	Women, higher concern [4,23,55,57,58,59,60,61,62,63]	Women, lower consumption [40,64,65,66,67,68,69,70,71,72]
Socio-demographic	Age	Mixed findings [23,58,59,73]	Mixed findings [43,60,72,74,75,76,77,78,79]
Socio-demographic	Education	Higher education, higher concern [58,59,60,73]	Higher education, lower consumption [40,58,74,75,78,80]
Socio-demographic	Income	Weak or mixed associations [73,81]	Mixed findings [9,10,60,72,77,79]
Socio-demographic	Household size	Limited evidence	Larger households, higher consumption [22,60,72,79,82]
Socio-demographic	Children in household	Limited evidence	No children, lower consumption [22,72,78,83]
Socio-demographic	Urbanization	Urban, higher concern [4,59,84,85]	Urban, lower consumption [4,75,76,79,86]
Animal-related	Pet ownership	Weak positive association [59,84,87,88]	Limited evidence
Animal-related	Farming experience	Often, lower concern [59,84,88,89]	Limited or unclear
Ideological	Religiosity	Often, lower concern [4,90,91,92]	Religion-specific effects [74]
Ideological	Political orientation	Left-wing, higher concern [93,94,95]	Right-wing, higher consumption [79,96,97,98,99,100]

Note: FAW = farm animal welfare. References are reported separately for each outcome variable.

Although the meat paradox has been widely discussed in the literature, less is known about which consumer groups differ simultaneously in FAW concern and dietary behavior and which background characteristics are associated with both outcomes within the same population. Addressing this gap may contribute to a better understanding of how concern for FAW is distributed across dietary groups.

In the present study, we examine whether FAW concern differs across dietary groups and which background factors are associated with both FAW concern and dietary self-classification. Identifying the consumer groups that differ in both outcomes is an important first step toward understanding the relationship between concern for farm animal welfare and dietary behavior. This study aims to elucidate the shared and distinct background factors associated with consumers’ FAW concern and diet. By examining the extent to which these outcomes are associated and linked to similar background characteristics, the study contributes to understanding how concern for farm animal welfare is distributed across dietary groups in contemporary society.

### Research Questions

1. Are there differences in the level of FAW concern between omnivores, flexitarians, vegetarians, and vegans?
2. What is the predictive power of socio-demographic, animal-related, and ideological background factors for both FAW concern and dietary behavior of omnivores, flexitarians, vegetarians, and vegans?

Definitions of FAW concern and FAW attitude vary in the literature. Some studies provide an explicit definition of FAW concern as an attitudinal evaluation of farm animal welfare, including ethical considerations and behavioral intentions [4,32,66], whereas FAW attitude is often operationalized as consumers’ perceptions and evaluative judgments of animal welfare, sometimes including willingness to pay for welfare-friendly products [24]. Other studies measure these concepts more implicitly, using surveys on perceptions, emotions, or purchase intentions without a clearly articulated definition [81,84,92]. In our study, FAW concern is conceptualized as an attitudinal construct, capturing a range of evaluations, including the perceived importance of farm animal welfare, the adequacy of current welfare standards, and the extent to which human practices, such as meat consumption, are seen as contributing to animal suffering. This conceptualization partially aligns with that of the Special Eurobarometer survey [1] which allows comparison of European data with the data of the study at hand.

## 2. Materials and Methods

### 2.1. Procedure

To examine the extent to which background factors are related to consumers’ self-reported FAW concern on the one hand and dietary self-classification pattern on the other, we conducted a survey among Dutch citizens in 2024.

### 2.2. Participants

A total of 9495 people were invited to participate in a survey from the ISO-20252:2019-certified panel of research agency Motivaction called “StemPunt” (over 70,000 active members) [101,102]. Of these, 2116 Dutch people aged 18 to 80 years old completed the full questionnaire (response rate of 0.22). Representativeness was ensured through sample selection procedures and post-stratification weighting calibrated to the Dutch adult population on age, gender, education, region, and ethnic background. Nevertheless, because information on individuals who declined participation was unavailable, potential non-response bias cannot be fully excluded. The weights were calibrated to match the Dutch adult population on five socio-demographic characteristics, age, gender, educational level, region (Nielsen classification), and ethnic background (European vs. non-European origin), using the Dutch “CBS Gouden Standaard” (“CBS Gold Standard”) benchmark, based on CBS microdata and maintained by Statistics Netherlands (https://www.cbs.nl/en-gb/figures/statline/information-about-statline, accessed on 28 May 2026). Population weights ranged from 0.67 to 2.42 (M = 1.00, SD = 0.44), indicating moderate variation in weighting adjustments.

Although the nominal sample size was N = 2116, weighting increases the variance of parameter estimates (design effect > 1), thereby reducing statistical efficiency. As a result, the effective sample size was Neff = 1777. The effective sample size reflects the amount of independent statistical information in the weighted data, providing the appropriate basis for statistical inference and power estimation.

### 2.3. The Questionnaire

The survey was completed between 3 and 13 December 2024. As a reward for participating, participants received savings points in the StemPunt savings system, which respondents can ultimately exchange for presents.

The questionnaire consisted of six blocks of questions (124 items in total). Only a small number of the items were relevant for this current study. Supplementary Materials Tables S1 and S2 list all relevant survey items and response options in both the original Dutch version and the English translation.

### 2.4. Outcome Variables

Diet was measured using a single self-classification item, asking respondents to select the dietary pattern that best described their eating habits. Similar self-classification measures are commonly used in research on meat consumption and meat reduction because they distinguish between broad dietary patterns that differ in the extent to which animal products are consumed [32,50]. For the purpose of the present study, which focused on broad differences in FAW concern between dietary groups, dietary self-classification provided a parsimonious and interpretable categorization of respondents’ eating patterns. However, such measures do not directly assess meat consumption frequency or quantity and may therefore not fully reflect actual dietary behavior. Consequently, the measure should be interpreted as reflecting self-reported dietary category rather than objectively measured meat consumption.

Participants were asked “Which of the following statements about how you eat applies to you best?” Four response options were accompanied by brief mouse-over explanations clarifying the meaning of each category (e.g., “flexitarian”: “you eat no meat for at least one or more days a week”).

The response options were

- “I eat flexitarian” (“you eat no meat for at least one or more days a week”);
- “I eat pescatarian” (“you eat no meat; but you do eat fish and shellfish”);
- “I eat vegetarian” (“you don’t eat meat or meat products, fish or shellfish”);
- “I eat vegan” (“you don’t eat meat, fish, shellfish, eggs, cheese, milk, or yogurt”);
- “I do not follow a special diet”;
- “Other, namely: …”;
- “I prefer not to say”.

The flexitarian category was intentionally defined broadly to capture the wider spectrum of consumers who reduce their meat consumption. This operationalization is broadly consistent with earlier Dutch research adopting an inclusive approach to meat reduction [103], while acknowledging that no universally accepted definition of flexitarianism exists in the literature [104].

Open-text responses under “Other, namely” were manually reviewed and reclassified if they corresponded to one of the predefined options. Two ambiguous descriptions and one empty description were coded as “I prefer not to say”. Respondents who indicated they did not follow a specific diet were labeled as “omnivores” (meat eaters).

FAW concern was assessed using a five-item scale. Three items were adapted from the Dutch version of the EU’s Special *Eurobarometer* “Attitudes of Europeans towards animal welfare 2023” questionnaire [1]. These items assessed (1) the importance of protecting farm animal welfare, (2) the belief that farm animal welfare should be better protected than it currently is, and (3) the importance of improving animal welfare in slaughterhouses. An example item is “How important do you think it is to protect the welfare of farm animals (livestock, e.g., pigs, cows, poultry, etc.) so that they have decent living conditions?” Respondents answered on a four-point scale ranging from 1 (“not important at all”) to 4 (“very important”), including the option “I don’t know”. To complement these items, we included two additional items capturing problem awareness and awareness of consequences, grounded in Schwartz’s Norm Activation Model [105], to assess the degree to which respondents (4) perceive farm animal welfare as currently insufficient and (5) believe that meat consumption contributes to animal suffering. Each of the two statements was rated on a six-point agreement scale, from 1 (“strongly disagree”) to 6 (“strongly agree”), and was linearly rescaled to match the 1–4 range of the first three items for comparability. The scale score was calculated as the mean across items. Together, the five items were intended to capture a broader construct of FAW concern, combining perceived importance of farm animal welfare with problem awareness and awareness of consequences regarding animal suffering. This conceptualization is broadly consistent with approaches in environmental psychology, where concern about societal issues is often operationalized using related but distinct components such as perceived importance, problem awareness, and awareness of consequences [103,106]. For descriptive interpretation, we categorized the mean scores into four concern levels using equal intervals across the theoretical range of the scale (1–4). The scores may be interpreted as follows: 1.00–1.75 (not concerned at all), 1.76–2.50 (low concern), 2.51–3.25 (moderate concern), and 3.26–4.00 (strong concern).

### 2.5. Background Factors

Socio-demographic variables were operationalized as categorical variables and dummy-coded for analysis. Age was initially collected as a continuous variable and subsequently recoded into four age groups (<35, 35–49, 50–65, and >65 years) to facilitate interpretation and comparison across life stages. Other socio-demographic variables were gender and global/geographical background (European vs. non-European, determined by the reported country of birth of the respondent and their parents), income (above average, average, below average), highest level of completed education (high, medium, low), and parental status (has children vs. no children).

Three variables were used to describe respondents’ geographic location.

Religion was categorized by self-reported religious affiliation (Christian, atheist, other, or prefer not to say).

Voting behavior was measured by asking the respondents which political parties they voted for during the Dutch parliamentary elections in November 2023. Based on classifications provided by Motivaction, responses were grouped into four categories: unknown (23%), left-wing, centrist, and right-wing.

To assess respondents’ personal experience and contact with animals, we included several items concerning exposure to animals. Respondents were asked whether they had visited livestock farms in the past two years and whether they had ever worked in livestock farming. Regular contact with animals was assessed with a multi-response item asking whether respondents frequently interact with different animal species in daily life. These variables were coded as four binary items indicating whether they frequently interacted with animals in general, with pets, with livestock, and with other animals.

### 2.6. Statistical Analyses

Statistical analyses were performed using Python (python.org 3.10.16) and the libraries Numpy 2.1.3 [107], Pandas 2.2.3 (https://doi.org/10.5281/zenodo.13819579), Scikit-learn 1.6.1p [108], SciPy 1.15 [109], and Statsmodels 0.14.4 [110]. The matplotlib library 3.10.1 [111] was used to produce the figures.

All statistical analyses were conducted using population weights rather than raw counts to ensure representativeness of the Dutch adult population. Supplementary Material Table S3 presents the sample characteristics for all background factors, including unweighted frequencies, weighted frequencies, weighted percentages, effective sample sizes, and weighted percentages per background variable category (e.g., age group, gender, education).

### 2.7. Bivariate Association Analysis

We examined weighted bivariate associations between each background factor and two outcomes: expressed FAW concern (continuous) and dietary group (categorical, five categories). Associations with FAW concern were assessed using weighted η^2^ from one-way analyses of variance, while associations with dietary group were assessed using bias-corrected Cramér’s V based on weighted contingency tables. For each association, 95% confidence intervals were estimated using nonparametric bootstrap resampling. No null-hypothesis significance tests were performed; results were interpreted based on effect sizes and confidence intervals.

### 2.8. Random Forest Analysis

A Random Forest analysis (RF) was used to assess how well the studied background factors predict FAW concern and dietary self-classification, allowing for complex, potentially non-linear relationships and interactions among background factors (see Appendix A for technical details of model specification, tuning, and validation). RF analysis wass used because it is well-suited for high-dimensional data, it is relatively robust to collinearity, and it is generally resistant to overfitting through ensemble averaging. Moreover, RF analysis has demonstrated good predictive performance in prior consumer behavior research on meat consumption and dietary choice [33]. We used an RF classifier for self-classified dietary behavior and an RF regressor for FAW concern.

Throughout this article, the term ‘”prediction” is used in the technical sense commonly employed in statistics and machine learning, referring to the extent to which observed characteristics can improve inference about an outcome variable. It does not imply causal relationships or forecasts of future behavior. Given the cross-sectional nature of the data, the findings should be interpreted in terms of associations and relative variable importance rather than causality.

To assess the contribution of individual background factors, both Gini importance scores (mean decrease in impurity) and SHAP (SHapley Additive exPlanations) values were used. Higher Gini scores indicate greater overall predictive relevance but do not convey the direction of an association (positive or negative). Therefore, SHAP values were used to complement the Gini scores. SHAP values quantify, at the individual-observation level, how the presence or absence of a given categorical characteristic increased or decreased the predicted likelihood of a specific outcome (FAW concern level or dietary group). This allowed interpretation of both the direction and magnitude of each variable’s contribution to model predictions. In the graphs with the SHAP values, each dot represents one respondent. The horizontal position of a dot indicates the contribution of a background factor to the predicted level of FAW concern (positive values indicate higher predicted FAW, negative values indicate lower predicted FAW). Dot color represents whether a feature was present or not for the respondent.

To ensure transparency, all data preparation and statistical analyses were conducted using reproducible Python scripts. The full analysis pipeline, including annotated scripts and documentation, is publicly available on the Open Science Framework (OSF) (https://osf.io/y4hmd/metadata/osf, accessed on 28 May 2026), enabling full reproducibility of all reported results.

## 3. Results

### 3.1. Farm Animal Welfare Concern

A total of 109 respondents (5.2%) did not answer more than one item on the five-item FAW scale. These were treated as missing values. With a Cronbach’s alpha of 0.85, the reliability of the scale is good [112]. Item-level descriptive statistics and additional scale diagnostics, alpha-if-item-deleted statistics, and confirmatory factor analysis results are reported in Appendix B. The weighted mean is 2.99 (“moderately concerned”) with a standard deviation of 0.63. This indicates that, on average, respondents report moderate concern for FAW, with limited dispersion around the mean, suggesting relatively homogeneous attitudes rather than strong polarization within the sample.

### 3.2. Dietary Self-Classification

The group that preferred not to disclose its diet was relatively small (effective *n* = 52, 3.5%). Most respondents classified their diet as omnivorous (effective *n* = 969, 55.6%), followed by flexitarians, who consumed no meat for at least one day a week (effective *n* = 582, 29.3%). The vegetarian and vegan respondents were clustered into a single category labeled veg*n (effective *n* = 139, 8.7%). This was due to the small number of vegans in the weighted sample (effective *n* = 27, 2,0%) and the overlap with vegetarians (effective *n* = 113, 6.7%) regarding the abstention from meat. Although relatively few respondents reported being pescatarian (effective *n* = 46, 2.8%), this group was included as a separate category in the bivariate analysis but excluded from the RF because of the limited sample size.

#### 3.2.1. Results of Bivariate Association Analysis

##### Associations Between Diet and FAW Concern

A strong association was observed between dietary self-classification and FAW concern (η^2^ = 0.190), representing a large effect size according to Cohen’s (1988) benchmarks [113]. Veg*n respondents expressed the highest FAW concern (M = 3.52, strong concern). Pescatarian (M = 3.28) and flexitarian respondents (M = 3.24) expressed almost similar moderate to strong concern. The mean values of expressed FAW concern of omnivores (M = 2.77) and respondents who preferred not to disclose their diet (M = 2.77) are lower than those of flexitarians, pescatarians and veg*ns.

##### Associations Between Background Factors, FAW Concern, and Diet

Weighted bivariate associations were examined between each background factor and both FAW concern and diet. Supplementary Material Table S3 presents descriptive statistics for FAW concern and dietary self-classification by background factors (weighted FAW concern means, diet percentages, and 95% CIs for all categories).

##### Associations Between Background Factors and FAW Concern

**Figure 1:**
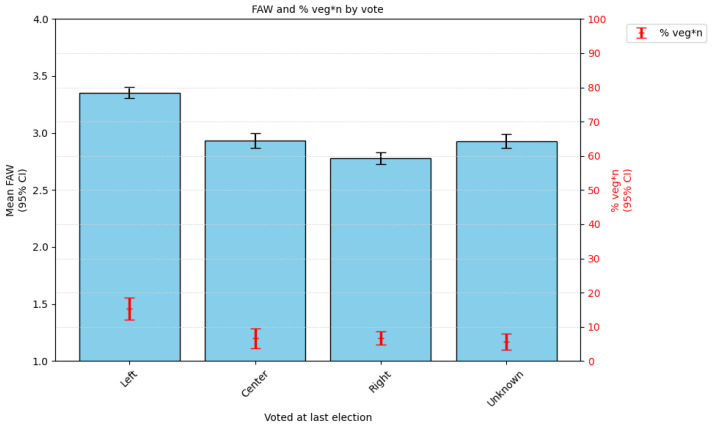
Associations between political orientation and FAW concern score (mean plus 95% CI in blue bars), and between political orientation and veg*n diet (point estimates with 95% CI in red).

Political orientation showed the strongest association with FAW concern (η^2^ = 0.135): left-wing voters reported the highest FAW concern (*M* = 3.35), followed by centrists (*M* = 2.93) and right-wing voters (*M* = 2.78; see Figure 1). Smaller differences were observed for religion (η^2^ = 0.029; atheist 3.10 vs. Christian 2.87), gender (η^2^ = 0.021; women 3.08 vs. men 2.90), regular contact with animals in general and with pets in particular (each η^2^ = 0.021, those with regular contact reporting higher FAW), household size (η^2^ = 0.012; one person 3.07 vs. three or more persons 2.91) and education level (η^2^ = 0.010; high 3.07 vs. low 2.90).

The smallest associations (η^2^ ≤ 0.010) were observed for having ever visited a livestock farm, ethnic background, rurality, age, regular contact with livestock, work experience with livestock, geographical region, parental status, and household income.

##### Associations Between Background Factors and Diet

Political orientation shows the strongest association with diet (Cramér’s V = 0.21), followed closely by religion (Cramér’s V = 0.20). Veg*n prevalence was substantially higher among left-wing voters (15.3%) than among non-left-wing voters (5.6–6.7%; see Figure 1, right *y*-axis). Similarly, veg*n prevalence was higher among non-Christian religious participants (17.5%) and atheists (9.4%) than among Christians (4.3%).

Associations were smaller for prior livestock work experience (Cramér’s V = 0.15; veg*n: 17.3% with experience vs. 8.1% without experience) and education level (Cramér’s V = 0.14; veg*n: 11.4% for high education vs. 7.4 and 7.5 for medium and low education).

Associations with diet were smallest (Cramér’s V ≤ 0.09) for gender, age, children in the household, household size, income, region, contact with animals, livestock farm visits, and rurality.

#### 3.2.2. Results of Random Forest Analysis

##### Predicted FAW Concern

The RF for FAW concern showed modest predictive performance overall, with an R-squared of 0.19 on the test set and 0.32 on the train set. For more details on the overall performance, see Appendix A. Left-wing voting, with a Gini importance of 0.36, is the strongest of the background predictor of FAW concern in the RF, far exceeding the Gini scores of all other background factors. Left-wing voting is followed by having regular contact with animals (Gini score 0.06) and gender (0.05). Associations with other background factors are relatively weak with Gini scores ≤ 0.03.

**Figure 2:**
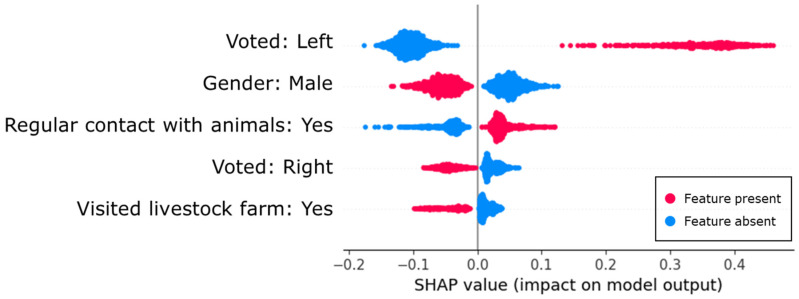
RF SHAP values of background factors (features) for FAW concern.

Figure 2 shows the SHAP values for the five most important background factors. Consistent with the Gini results, left-wing voting contributed most clearly to higher predicted FAW concern. The following features also contributed to higher predicted FAW concern in the RF analysis, albeit to a lesser extent: not being male (hence being female), having regular contact with animals and not voting for right-wing parties. Having ever visited a livestock farm slightly negatively contributed to expressed FAW concern. In other words, higher FAW concern was more often observed among respondents who voted left-wing, were female, had regular contact with animals, did not vote right-wing, and had not visited a livestock farm.

##### Predicted Dietary Self-Classification

**Figure 3:**
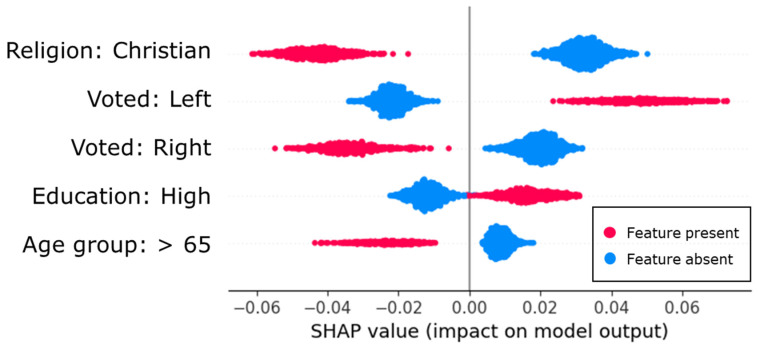
RF SHAP values of background factors (features) for self-identified veg*n diet.

**Figure 4:**
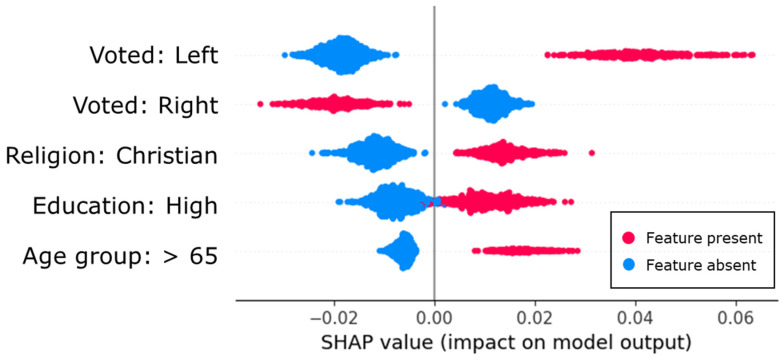
RF SHAP values of background factors (features) for self-identified flexitarian diet.

**Figure 5:**
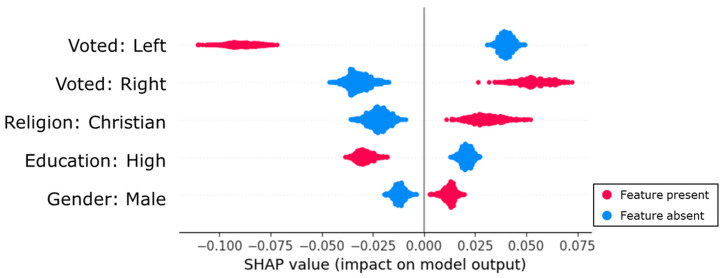
RF SHAP values of background factors (features) for self-identified omnivorous diet.

Overall, the background factors explain only a modest portion of the dietary self-classification, with an accuracy of 0.5 in the test data and 0.64 in the train data (see Supplementary Material Table S3 and Appendix A for all details). As with the FAW model, the relative predictive value of the background factors on the outcome variable self-classified dietary behavior is analyzed using both Gini scores and SHAP values. Political orientation, religious affiliation, and gender are consistently the most important predictors according to Gini importance scores. Gini scores are 0.12 for left-wing voting, 0.07 for Christian religion, 0.06 for identifying with another religion (non-Christian), and 0.04 for high education. All other factors have Gini scores of 0.03 or lower, suggesting limited predictive value. Figure 3, Figure 4 and Figure 5 present for each diet group, more specifically, the SHAP values of the background factors.

#### 3.2.3. Self-Identified veg*n Diet

In the RF, SHAP values indicated that the five strongest contributors to predicting a veg*n diet were (1) not identifying as Christian, (2) left-wing voting, (3) not voting right-wing, (4) higher educational attainment, and (5) not being 65 or older.

##### Self-Identified Flexitarian Diet

In the RF, predictions of self-identified flexitarian diet were primarily driven by political orientation, religion, education and age. Within the model, left-wing voting showed relatively strong positive SHAP contributions to self-identified flexitarian diet, whereas right-wing voting contributed negatively. Identifying with Christian religion contributed positively. Higher education and being over 65 years of age increased the predicted probability of a flexitarian diet as well.

##### Self-Identified Omnivorous Diet

In the RF, predictions of self-identified omnivorous diet were primarily driven by political orientation, religion, education and gender. Not voting left-wing, right-wing voting, Christian religion and being male showed relatively strong positive SHAP contributions to self-identified omnivorous diet. Higher education had a slight negative predictive value for a self-identified omnivorous diet.

## 4. Discussion

This study examined how various socio-demographic, animal-contact-related, and ideological background factors are associated with both FAW concern and dietary self-classification. It aimed to clarify the shared and distinct predictors of FAW concern and self-reported meat consumption. What follows in this discussion is that we first address differences in FAW concern between omnivores, flexitarians, vegetarians and vegans. Then the predictive value of socio-demographic, animal-related, and ideological background factors for FAW concern and diet are examined. Consecutively, we will compare our dietary behavior and FAW results with those of other studies. The section concludes with limitations and directions for future research.

The first research question asked whether there are differences in FAW concern levels among omnivores, flexitarians, vegetarians and vegans. Our results show meaningful differences in FAW concern across these groups. On average, veg*ns express the highest concern for FAW, with a mean score of 3.52 at a scale from 1 (lowest) to 4 (highest). Interestingly, pescatarians and flexitarians limit their consumption of red meat and poultry in different ways and express almost identical levels of FAW concern (M = 3.28 and M = 3.24 respectively). These findings should be interpreted in light of the fact that diet was assessed through dietary self-classification rather than direct measures of meat consumption. Possible explanations for the pescatarian’s moderate–strong FAW concern combined with fish consumption may be a lack of knowledge or experience regarding the poor welfare of fish in fisheries, or the belief that fish are less sentient animals [114,115]. The moderate–strong FAW concern expressed by flexitarians do not necessarily translate into overall reduced meat consumption [51], since in the study at hand only consumption frequency has been reported. Furthermore, the broad definition of flexitarianism used in this study likely encompasses consumers with substantially different levels of meat consumption. Consequently, the observed FAW concern among flexitarians should be interpreted as reflecting an average across a heterogeneous group, and direct comparisons with studies using different definitions of flexitarianism should be made with caution.

Although FAW concern is clearly associated with dietary self-classification, the findings also reveal a notable pattern at population level (see Supplementary Material Table S3). Approximately 37% of the total population reported moderate to strong FAW concern while simultaneously identifying as omnivorous, whereas approximately 26% reported similar levels of FAW concern while identifying as flexitarian. These findings suggest that concern for FAW and meat consumption are not mutually exclusive and may coexist at the population level. The relationship between FAW concern and dietary behavior is more complex than a straightforward correspondence between concern and diet. The persistence of relatively high FAW concern among some self-identified omnivores may reflect a variety of psychological processes discussed in the meat paradox literature, including cognitive dissonance reduction, moral disengagement, and other rationalization strategies [116]. However, because the present study assessed self-classified dietary patterns rather than actual meat consumption and did not directly measure these psychological mechanisms, no conclusions can be drawn regarding the processes underlying this apparent discrepancy.

The second research question focused on the predictive value of socio-demographic, animal-contact-related, and ideological background factors for FAW concerns of omnivores, flexitarians, vegetarians and vegans.

Before discussing the findings, it is important to note that this study is based on cross-sectional data. As such, the associations between background factors and behaviors, such as dietary self-classification or concern about FAW, should not be interpreted as causal relationships. For example, when political orientation is identified as a relatively strong predictor of dietary self-classification, this reflects a significant association rather than a causal effect.

Overall, biological and socio-demographic background factors showed only limited and inconsistent associations with FAW concern and dietary self-classification. Age is not associated with FAW concern and shows no clear association with dietary self-classification groups, which contrasts with findings from other studies [22,60]. In the model, being over 65 was associated with a slightly lower likelihood of being veg*n and a slightly higher likelihood of being flexitarian. This weak association suggests that, within the Dutch context, generational differences may play a less pronounced role than often assumed.

Gender shows a weak to moderate predictive value for FAW concern, with women expressing greater concern than men, consistent with prior studies. This difference does not translate into dietary behavior. These findings do not support statements [64,65] that gender (femininity) is the most significant predictor of vegetarianism, nor that masculinity is the key predictor of meat consumption. Similarly, most socio-demographic variables, including income, region, and origin, show little to no association with FAW concern or diet.

Education is the main exception, as higher education is associated with a greater likelihood of meat-reduced diets, although its association with FAW concern remains weak. This pattern suggests that other factors such as health concerns, social norms and habits may be more important than prosocial concerns about FAW in reducing meat consumption among higher-educated citizens.

Household composition also shows limited explanatory value. Although larger households are weakly associated with lower FAW concern, no clear relationship with self-classified dietary behavior is observed. This contradicts other studies [22,78] reporting that households with children tend to consume more meat. Taken together, these findings indicate that commonly studied socio-demographic factors have only limited predictive power for explaining differences in FAW concern and self-classified dietary behavior.

In our study, animal-contact-related background factors show small and inconsistent associations with FAW concern. Regular contact with animals is weakly associated with higher FAW concern, whereas prior farm visits are associated with slightly lower concern. No significant associations were found between animal-contact-related background factors and dietary behavior. These findings align with previous studies listed in Table 1.

Prior experience with farm animals is associated with lower levels of FAW concern as has been reported previously [88,117,118]. These findings suggest that different forms of animal contact may have divergent effects.

One possible explanation is that everyday contact with pets may strengthen emotional engagement and concern, whereas having regular contact with livestock or work experience in the meat-producing sector may normalize existing production systems and reduce perceived welfare concerns. However, given the weak effects observed, these factors appear to play only a minor role in explaining variation in FAW concern and dietary behavior.

In contrast to the limited role of socio-demographic and animal-contact-related factors, ideological background factors, particularly political orientation, emerge as the most consistent and substantive predictors of both FAW concern and dietary self-classification. Left-wing voting respondents reported the highest FAW concern and most likely reported a veg*n diet, while right-wing voting shows the opposite pattern. These findings confirm other studies showing that left-wing voting is associated with greater FAW concern and lower meat consumption, while right-wing voting is associated with lower FAW concern and higher meat consumption [92,94,95].

Religious affiliation shows a more moderate but consistent pattern. Christian religious affiliation is associated with lower FAW concern and a higher likelihood of self-identified omnivorous or flexitarian diets. This may be explained by certain Christian fundamentals, such as stewardship over animals [119]. Identifying with atheism or a non-Christian religion modestly predicts higher FAW concern, which aligns with findings in other studies of greater FAW concern among non-religious individuals [91].

Notably, political orientation and religious affiliation showed consistent associations with both FAW concern and dietary self-classification across the conventional analyses and the Random Forest models. Political orientation displayed the strongest associations across both analytical approaches. The convergence of findings strengthens confidence in the robustness of these observed relationships.

Taken together, these findings suggest that differences in FAW concern and dietary self-classification are more consistently associated with ideological orientations than with animal-contact-related and traditional socio-demographic background factors. One possible explanation is that political and religious orientations reflect broader differences in values, moral beliefs, and views on human–animal relationships. Research in political psychology suggests that political preferences are partly rooted in basic human values (Schwartz, 2014; Feldman, 2003 [120,121]) and moral intuitions (Graham et al., 2009 [122]), which may also shape attitudes toward farm animal welfare and dietary behavior. While factors such as gender and education show some associations, their predictive power is limited compared to that of political orientation. This pattern is in line with previous research showing that political orientation is systematically related to FAW concern and meat consumption (Table 1). However, these findings are based on cross-sectional associations and should not be interpreted as causal relationships. Socio-demographic factors may, at least partly, shape individuals’ ideological orientations, which in turn are associated with FAW concern and dietary self-classification. Although weaker in magnitude, factors such as gender, education level, and animal contact still show modest but significant associations with both outcomes.

A key strength of this study is the large sample size (N = 2116), which enhances statistical power and enables meaningful comparisons across diet groups and FAW concern. The sample is representative of the Dutch adult population, reflecting its demographic characteristics (age, gender, education level, region, and origin) and the interactions among these variables, as shown in recent national statistics (e.g., Central Bureau of Statistics of the Netherlands, 2021 [102]). Stratified sampling techniques were used to ensure adequate representation of key demographic subgroups. In comparison to other studies, the 7.7% self-identified veg*ns and 32.8% self-identified flexitarians in this study are within the range of percentages commonly reported [40,44,45,46]. Similarly, the 2.6% of self-identified pescatarians in our study align with the percentages in other European countries [43]. However, the value of 32.8% of self-identified flexitarians in our study is 10% lower than the 43% reported in previous research in 2019 [40]. This discrepancy may be explained by differences in definitions and classification criteria for flexitarians. However, our operationalization “Eating no meat for at least one or more days a week” corresponds to light vegetarian segment (eating meat with their hot meal an average of 5 to 6 times a week) reported by Verain and coworkers [29,40]. In addition, self-identified flexitarians may exhibit behaviors like those of self-identified omnivores. It means that consumers with similar dietary behavior may be (self) classified differently in various studies [47].

Furthermore, some studies focus on broader animal welfare attitudes (AWA) rather than specifically on farm animal welfare (FAW). Inconsistencies in definitions and operationalizations of constructs can lead to varying results across diet groups. Differences in country, sample selection, and timing also affect comparability, with older studies potentially reflecting outdated views as public interest in FAW grows.

Another reason for the observed discrepancies in FAW concern across studies may relate to differences in methodology. The Special Eurobarometer 2023 and our study used three similar items to assess FAW attitudes. The results of our study and those of Special Eurobarometer 2023 reveal notable differences in responses. For example, 38% of respondents in our study consider the welfare of farmed animals to be “very important” (Q2), compared with 57% of the Dutch respondents in the Eurobarometer. Similarly, the perception of whether FAW should be better protected (Q3) was expressed by 28% of our respondents, whereas 45% of Eurobarometer Dutch respondents strongly agreed. Regarding the importance of improving welfare in slaughterhouses (QC10), 41% of our respondents deemed it “very important,” compared with 67% of the Dutch respondents in the Eurobarometer. Several factors may have contributed to the variation in responses. The placement of questions in surveys can influence respondents’ answers. In the Special Eurobarometer, the question about the welfare of animals in slaughterhouses (QC10) was the tenth question, following several animal welfare-related questions, which may have allowed respondents to reflect more on the issue after considering other topics. In contrast, our study asked this question early in the survey, which may have influenced respondents’ initial engagement with the subject. Moreover, the data-collection method may have influenced the results. The Special Eurobarometer conducted face-to-face interviews, which may have encouraged socially desirable responses. Previous methodological research has shown that interviewer-administered surveys are generally more susceptible to social desirability bias than self-administered web surveys, particularly when questions concern socially sensitive or morally salient topics [123,124]. Research comparing survey modes further suggests that web surveys may elicit more accurate reporting of sensitive attitudes and behaviors by reducing interviewer effects and perceived social pressure [115]. Respondents might therefore have felt compelled to provide socially acceptable answers in a face-to-face setting, whereas our online survey, offering greater anonymity, may have reduced these biases. This may explain the lower levels of FAW concern observed in our study compared to the Eurobarometer. Consequently, direct comparisons between the absolute levels of FAW concern reported in both surveys should be interpreted with caution.

Sample size may also have influenced the results as the Special Eurobarometer included 1001 Dutch respondents while our sample was 2116 respondents. However, it is unlikely that sample size explains the substantial difference between the results of the Special Eurobarometer and our data.

### 4.1. Future Research

One of the key findings of the present study is that political orientation emerged as the background factor with the highest relative predictive importance for both FAW concern and dietary self-classification. This raises an important question regarding the nature of this association. Political orientation itself is unlikely to be a causal driver of dietary behavior; rather, it may reflect underlying psychological or social mechanisms. From a political psychology perspective, the strong association between political orientation and diet likely reflects underlying values and moral convictions. Political preference is known to correlate with concern for disadvantaged groups [93] and individuals’ basic value orientations are linked to political preference and voting behavior over time [125]. Moreover, empirical evidence shows that political differences (e.g., liberal versus conservative orientations) are systematically related to distinct moral foundations [53,122,126,127]. These value orientations may help explain why meat consumption remains self-evident for some individuals despite FAW concern, while for others reducing or avoiding meat is considered obvious or morally necessary.

In addition, group membership and the motivation to maintain a positive social identity influence how individuals align their beliefs and behaviors with those of their reference groups [119,128]. Political orientation can therefore be understood not only as an ideological position but also as an expression of identity embedded in a broader social environment. Group identity and socialization processes develop across the lifespan, with childhood and adolescence often described as crucial developmental stages. During these periods, social norms surrounding meat consumption and attitudes toward animals may become internalized. The social environment may thus play a substantial role in shaping both dietary behavior and FAW attitudes.

Future research should therefore move beyond traditional demographic background factors and focus more explicitly on human value orientations, moral foundations, identity processes, socialization mechanisms social and personal norms, habits and individuals’ self-efficacy to change dietary behavior. Such approaches may help explain why some individuals reduce or abstain from meat consumption in response to FAW and other moral concerns while others maintain established dietary practices despite expressing similar concerns. Together, these factors may provide a more comprehensive understanding of how moral concerns are translated into diet and other forms of prosocial behavior.

### 4.2. Limitations

This study provides valuable insights but also faces several limitations. First, although the aim was not to quantify total meat intake, the method used to assess dietary patterns presents a limitation. Dietary self-classification was assessed using a single self-reported item and lacked additional measures to provide a more detailed picture of respondents’ dietary behaviors. Moreover, because diet was assessed through self-classification rather than direct measures of meat consumption frequency or quantity, the results should be interpreted as reflecting dietary self-classification categories rather than objectively measured consumption behavior. The study also used a broad classification for flexitarians, which may mask variation in meat consumption within this group and complicate comparisons with studies using different operational definitions of flexitarianism [104]. A more detailed scale, such as the Meat Consumption Scale (MCS) [129], could offer a more fine-grained assessment in future research.

Second, the study does not differentiate between various types of meat, even though FAW concerns may vary across species, such as between farm animals and fish [115].

Third, the study relied solely on explicit survey measures, which may be subject to social desirability bias, particularly given the moral sensitivity of FAW. Moreover, explicit responses may not fully reflect underlying attitudes, as they can diverge from implicit evaluations [130] and may overestimate the importance of FAW concern in the study [12,79].

Finally, although a key strength of this study is the large and population-weighted sample, subgroup sizes for specific dietary categories were relatively small. Self-identified vegetarian and vegan respondents were therefore combined into a single category, and pescatarians could not be retained in all analyses. This limited the ability to examine differences between more specific dietary groups, such as self-identified vegetarians and self-identified vegans.

## 5. Conclusions

Clear differences in FAW concern were observed between the three self-identified dietary groups. Veg*ns reported the highest levels of FAW concern, followed by flexitarians, while omnivores expressed the least FAW concern. This pattern suggests that FAW concern is systematically associated with dietary behavior, with veg*ns being the most concerned and omnivores the least. However, despite these systematic differences across groups, at population level, the overall association between FAW concern and reduced meat consumption was limited. These findings are consistent with what is often described as the “meat paradox” [19] referring to the fact that the large majority people who morally care about animals and do not want them to suffer harm maintain a diet that requires animals to be killed and/or usually suffer. The results suggest that this discrepancy is present at the population level and structured by ideological orientations, reflecting broader ideological differences within society.

Ideological background factors, particularly political orientation and, to a lesser extent, religious affiliation, are most strongly associated with both FAW concern and diet. These ideological background factors have higher relative predictive importance than traditional socio-demographic or animal-related background factors. Taken together, these findings suggest that ideological orientations may be more informative than traditional socio-demographic characteristics for understanding differences in FAW concern and dietary behavior.

Overall, these findings suggest that interventions aimed at reducing meat consumption or promoting animal-friendly food choices may benefit from taking consumers’ ideological orientations, as well as differences in these orientations across consumer groups, into account. The relatively limited association between FAW concern and reduced meat consumption indicates that raising concern alone is unlikely to be sufficient to achieve behavioral change.

## Data Availability

The dataset and Python scripts used for data preparation and statistical analysis are publicly available on the Open Science Framework (OSF) repository (https://osf.io/y4hmd/metadata/osf, accessed on 28 May 2026), enabling full reproducibility of all reported results.

## Supplementary Materials

The following supporting information can be downloaded at https://www.mdpi.com/article/10.3390/ani16132110/s1, Supplementary Material Table S1: Table with all variables constructing farm animal welfare concern; Supplementary Material Table S2: Table with all questions for the background variables; Supplementary Material Table S3: Descriptive statistics of FAW concern and dietary self-classification by background characteristics (with 95% CIs).

## Appendix A. Hyperparameter Grid and Selected Model Configuration for the Random Forest Analysis

A Random Forest analysis (RFA) was used because of its ability to handle high-dimensional data with complex interactions and non-linear relationships, while being robust to multicollinearity and overfitting. Moreover, RFA provides meaningful estimates of variable importance, which aligns well with the aim of the study to explore the relative predictive contribution of each background variable.

We used the scikit-learn library (v1.6.1) for RFA. We used a Random Forest classifier for prediction of dietary group membership and a Random Forest regression for prediction of FAW concern (thus substituting regression trees for classification trees). The classification analysis was limited to the three larger dietary groups that were of main interest with respect to meat consumption: omnivores, flexitarians, and veg*ns. The pescatarian group was too small for separate analysis. Hence, pescatarians were reclassified as flexitarians (cf. Peri et al., 2025 [33]).

All predictive variables were categorical and were one-hot encoded prior to modeling, except for variables with only two categories, which were dummy-coded for enhanced clarity in the results.

The dataset was randomly split into a training set (70% of observations) and a test set (30%). Hyperparameters were optimized via grid search with three-fold cross-validation. The parameter grid and selected model configurations are described below. Population weights were incorporated into both models using the sample-weight parameter.

### Appendix A.1. Predicted Perceived Importance of Farm Animal Welfare

To investigate how well background variables predict individual differences in the perceived importance of farm animal welfare (FAW), we trained a Random Forest regression model. The model achieved an R-squared of 0.19 on the test set, indicating modest predictive performance. The R-squared on the training set was higher (0.32), suggesting some degree of overfitting.

	**Regression**	**Classifier**
n_estimators	[100, **200**]	[100, **200**]
max_depth	[None, **5**, 10]	[None, **5**, 10]
min_samples_split	[2, **5**]	[**2**, 5]
min_samples_leaf	[1, **2**]	[1, **2**]
Note: Bold numbers indicate the selected model configuration.		

### Appendix A.2. Predicted Diet Classification

For the classification model, the class-weight parameter was set to “balanced” to mitigate the strong class imbalance in the diet variable. Although this setting decreases model accuracy, it ensures that errors in predicting the small group of veg*ns are sufficiently weighted, preventing the model from defaulting to the majority classes (e.g., omnivores, flexitarians).

In the classification model, where both sample weight and class weight were applied, the final observation weights were computed as the product of both components.

A Random Forest classifier with balanced class weights was trained to predict dietary group membership among omnivores (58%), flexitarians (34%), and veg*ns (9%). The model achieved an accuracy of 0.64 on the training set and 0.50 on the test set, indicating modest predictive power. Performance differs across diet group. The classifier performed best for omnivores, correctly identifying most true cases (recall = 0.70) while keeping a relatively high proportion of its omnivore predictions correct (precision = 0.64). In contrast, fewer flexitarians and veg*ns were detected: recall was below 0.4 for both groups, and precision was particularly low for veg*ns (0.13). The F1 score, which combines precision and recall into a single measure, averaged across classes (macro F1) was 0.56 on the training set and 0.38 on the test set, suggesting slight overfitting. Precision, recall, and F1 scores per class are shown in the table below.

**Dietary Group**	**Precision**	**Recall**	**F1-Score**
Omnivores	0.64	0.70	0.67
Flexitarians	0.42	0.22	0.29
Veg*ns	0.13	0.33	0.19

### Appendix A.3. Confusion Matrix Random Forest Classifier

The confusion matrix clarifies how misclassifications are distributed across diet groups. Omnivores were classified most reliably (237/338 correct), with remaining omnivores more often misclassified as flexitarian (52) than as veg*n (49). Flexitarians were more frequently assigned to the omnivore class (111) and to veg*n class (55) than correctly identified as flexitarian (46). Veg*n was the hardest class to predict: most true veg*ns were misclassified as omnivore (21) or flexitarian (11), and veg*n predictions were often false positives (only 16 of 120 veg*n predictions are correct). Overall, misclassification tends to shift cases toward the majority classes, consistent with the low recall for veg*ns and flexitarians.

Example of Gini importance scores of features in the Random Forest Analysis for diet:

**Feature**	**Importance**
voted_label_Left	0.12
voted_label_Right	0.07
religion_rec_label_Christian	0.07
religion_rec_label_Other	0.06
education_label_High	0.04
origin_label_Non-European	0.03
age_groups_<35	0,03
gender_label_Male	0.03
education_label_Low	0.03
education_label_Medium	0.03
Nielsen6_label_North	0.03
age_groups_>65	0.02
salary_cat_label_Above average	0.02
household_size_rec_label_3 or more persons	0.02
regular_contact_with_animals_label_yes	0.02
Nielsen6_label_East	0.02
household_size_rec_label_2 persons	0.02
voted_label_Unknown	0.02
religion_rec_label_Atheist (non-religious)	0.02
Nielsen6_label_West	0.02
parental_status_label_Parent	0.02
salary_cat_label_Below average	0.02
raised_rural_label_Yes	0.02
parental_status_label_No parent	0.02
visited_livestock_farm_label_Yes	0.02
household_size_rec_label_1 person	0.02
age_groups_50-65	0.02
salary_cat_label_Average	0.02
salary_cat_label_Unknown	0.02
voted_label_Center	0.01
worked_livestock_farm_label_Yes	0.01
regular_contact_with_pets_label_yes	0.01
age_groups_35-49	0.01
regular_contact_with_livestock_label_yes	0.01
regular_contact_with_other_animals_label_yes	0.01
Nielsen6_label_South	0.01
Nielsen6_label_Suburban municipalities	0.01
Nielsen6_label_3 major cities	0.01
religion_rec_label_I prefer not to say	0.01
parental_status_label_Unknown	0.00

## Appendix B. Farm Animal Welfare Scale Validation

Internal consistency of the FAW scale is good (Cronbach’s α = 0.852). Item-level diagnostics indicates that all items contributed positively to reliability, as removal of any single item reduces or negligibly changes α. The weakest contributor was E1_2 (α-if-deleted = 0.847). Confirmatory factor analysis of a single-factor model yields adequate convergent validity (AVE = 0.54) and composite reliability, but marginal structural fit (CFI = 0.84), suggesting the items do not fully conform to a unidimensional construct structure. This is further supported by the observation that the A-item subscale (α = 0.832) and E-item subscale (α = 0.794) each demonstrate strong internal consistency independently, indicating the presence of two item clusters within the broader construct. The scale is valid as a unidimensional scale for scoring purposes, but a latent substructure appears to exist.

**Model**	**Cronbach Alpha**
full FAW scale	0.852
without A2_processed	0.825
without A3_processed	0.801
without A4_processed	0.825
without E1_1_processed	0.808
without E1_2_processed	0.847
FAW based on A2, A3, A4 only	0.832
FAW based on E1, E2 only	0.794
