# Supplementary material for: Farm Animal Welfare on the Mind and Meat in the Diet: Who Are These Consumers?
Source: Animals (Basel). 2026 Jul 7;16(13):2110. doi: 10.3390/ani16132110 (PMC13359821; doi:10.3390/ani16132110)
Supplement: Supplementary file 1 [file animals-16-02110-s001.zip › animals-4382682-supplementary.pdf]

Table S1: Table with all variables constructing farm animal welfare concern (FAW concern)

**FAW concern scale (ENG)**	**FAW concern scale (NL)**	**Response options (ENG)**	**Response options (NL)**
A2 How important is it according to you to protect the welfare of farm animals (livestock, for example pigs, cows, poultry etc.) so that they have decent living conditions?	A2 Hoe belangrijk is het volgens jou om het welzijn van landbouwhuisdieren (vee, bv. varkens, koeien, gevogelte, enz.) te beschermen zodat ze fatsoenlijke leefomstandigheden hebben?	not important at all<br>not important<br>important<br>very important<br>I don't know	Helemaal niet belangrijk<br>Niet zo belangrijk<br>Wel belangrijk<br>Erg belangrijk<br>Weet niet
A3 Do you think the welfare of farm animals (livestock) in the Netherlands should generally be better protected than it currently is?	A3 Vind je dat het welzijn van landbouwhuisdieren (vee) in Nederland over het algemeen beter beschermd zou moeten worden dan nu het geval is?	no, definitely not<br>no, probably not<br>yes, probably<br>yes, definitely<br>I don't know	Nee, zeker niet<br>Nee, waarschijnlijk niet<br>Ja, waarschijnlijk<br>Ja, zeker<br>Weet niet
A4 How important do you think it is to improve the welfare of animals in slaughterhouses, for example through more official inspections, including the use of video surveillance?	A4 Hoe belangrijk vind je het dat het welzijn van dieren in slachthuizen wordt verbeterd, bijvoorbeeld door meer officiële controles, waaronder ook met videocamera's?	not important at all<br>not important<br>important<br>very important<br>I don't know	Helemaal niet belangrijk<br>Niet zo belangrijk<br>Wel belangrijk<br>Erg belangrijk<br>Weet niet
E1_1 To what extent do you agree or disagree with the following statements? Farm animals in the livestock industry (livestock, like cows, chickens and pigs) have a lack of welfare.	E1_1 In hoeverre ben je het eens of oneens met de volgende stellingen? In de vee-industrie hebben landbouwhuisdieren (vee, zoals koeien, kippen en varkens) gebrek aan welzijn.	strongly disagree<br>somewhat disagree<br>slightly disagree<br>slightly agree<br>somewhat agree<br>strongly agree	Zeer mee oneens<br>Redelijk mee oneens<br>Enigszins mee oneens<br>Enigszins mee eens<br>Redelijk mee eens<br>Zeer mee eens
E1_2 To what extent do you agree or disagree with the following statements? Meat consumption contributes to animal suffering	E1_2 In hoeverre ben je het eens of oneens met de volgende stellingen? Vlees eten draagt bij aan dierenleed.	strongly disagree<br>somewhat disagree<br>slightly disagree<br>slightly agree<br>somewhat agree<br>strongly agree	Zeer mee oneens<br>Redelijk mee oneens<br>Enigszins mee oneens<br>Enigszins mee eens<br>Redelijk mee eens<br>Zeer mee eens

Intern gebruik

Table S2: Table with questions and response options for all background variables

**Background factors (ENG)**	**Background factors (NL)**	**Questions (ENG)**	**Questions (NL)**	**Response options (ENG)**	**Response options (NL)**
Grew up in rural area	Opgegroeid platteland	F1 Did you grow up or live in a rural area?	F1 Ben je opgegroeid of heb je gewoond op het platteland?	Yes; No	Ja; Nee
Exposure to animals	Blootstelling aan dieren	F2 Have you visited a livestock farm in the past two years?<br>F3 Have you worked in livestock farming?<br>F4 Do you regularly have contact with animals in your daily life?	F2 Ben je in de afgelopen 2 jaar op een veehouderij geweest?<br>F3 Heb je in de veehouderij gewerkt?<br>F4 Heb je in jouw dagelijks leven regelmatig contact met dieren?	F2 Yes; No<br>F3 Yes; No<br>F4 No; Yes, with my own pets; Yes, with other people's pets; Yes, with livestock; Yes, with other animals.	F2 Ja; Nee<br>F3 Ja; Nee<br>F4 Nee; Ja, met mijn eigen huisdieren; Ja, met huisdieren van anderen; Ja, met landbouwhuisdieren; Ja, met andere dieren.
Religious background	Religieuze achtergrond	F6 Which religious background or beliefs describes you the best?	F6 Welke religieuze achtergrond of levensovertuiging beschrijft jou het beste?	Christian; Muslim; Atheist (not religious); Buddhist; Hindu; Winti; Other, (please specify; Prefer not to say.	Christelijk; Islamitisch; Atheïstisch (niet gelovig); Boeddhistisch; Hindoeïstisch; Winti; Anders, namelijk:; Zeg ik liever niet.
Voting behavior	Stemgedrag	F7 Which political party did you vote for in the last Dutch parliamentary elections?	F7 Op welke politieke partij heb je bij de laatste Tweede Kamerverkiezingen gestemd?	VVD; D66; PVV; CDA; SP (Socialistische Partij); GroenLinks/PvdA; Forum voor Democratie (FvD); Partij voor de Dieren; ChristenUnie; Volt; JA21; SGP (Staatkundig Gereformeerde Partij); DENK; NSC; 50PLUS; BBB; BIJ1; Other party; Blank vote; Prefer not to say; Don't know.	VVD; D66; PVV; CDA; SP (Socialistische Partij); GroenLinks/PvdA; Forum voor Democratie (FvD); Partij voor de Dieren; ChristenUnie; Volt; JA21; SGP (Staatkundig Gereformeerde Partij); DENK; NSC; 50PLUS; BBB; BIJ1; Andere partij; Blanco stem; Zeg ik liever niet; Weet niet.

Degree of urbanization Classification by Nielsen region. The Netherlands has five Nielsen districts.<br>Very urban Nielsen I: the 3 major cities plus peripheral municipalities;<br>Highly urban Nielsen II West: North Holland, South Holland and Utrecht (excluding the Nielsen I municipalities);<br>Moderately urban Nielsen III, North: Groningen, Friesland and Drenthe;<br>Little urban Nielsen IV East: Overijssel, Gelderland and Flevoland;<br>Non-urban Nielsen V, South: Zeeland, North Brabant and Limburg	Mate van stedelijkheid Classificatie volgens Nielsen regio.<br>Nederland heeft vijf Nielsen districten. Heel stedelijk Nielsen I: de 3 grote steden en de omliggende gemeenten;<br>Sterk stedelijk (West) Nielsen II: Noord Holland, Zuid Holland en Utrecht (exclusief de Nielsen I gemeenten);<br>Matig stedelijk (Noord) Nielsen III: Groningen, Friesland en Drenthe;<br>Weinig stedelijk (Oost) Nielsen IV: Overijssel, Gelderland en Flevoland;<br>Niet stedelijk (Zuid) Nielsen V: Zeeland, Noord-Brabant en Limburg	This is not included in the questionnaire	Dit is niet opgenomen in de vragenlijst		
Income	Inkomen	This is not included in the questionnaire	Dit is niet opgenomen in de vragenlijst		
Education	Opleiding	This is not included in the questionnaire	Dit is niet opgenomen in de vragenlijst		
Age	Leeftijd	This is not included in the questionnaire	Dit is niet opgenomen in de vragenlijst		
Gender	Geslacht	This is not included in the questionnaire	Dit is niet opgenomen in de vragenlijst		
Origin	Herkomst	European or non-European?	Europees of niet Europees?	European; Non-European	Europees; Niet-Europees
Household size	Huishoudgrootte	How many people are there in your household, including yourself?	Uit hoeveel personen bestaat uw huishouden, inclusief uzelf?	1 person; 2 people; 3 people; 4 people; 5 people; 6 or more	1 persoon; 2 personen; 3 personen; 4 personen; 5 personen;

				people; Prefer not to say.	6 personen of meer; Wil ik niet zeggen.
Amount of children in the household	Aantal kinderen in het huishouden	How many children do you have, including stepchildren and adopted children?	Hoeveel kinderen heeft u? Stiefkinderen en adoptiekinderen ook meegerekend kind(eren)	1; 2; 3; 4; 5; 6; 7; 8; 9; 10; 11; 12; 13; 14; 15; Unknown; No children.	1; 2; 3; 4; 5; 6; 7; 8; 9; 10; 11; 12; 13; 14; 15; Onbekend; Geen kinderen.

Table S3: Descriptive statistics of FAW concern and dietary self-classification by background characteristics (with 95% CIs).

**Variable**	**Category**	**N**	**Neff**	**Category %**	**mean FAW concern**	**% Omnivore**	**% Flexitarian**	**% Veg*n**	**% Vegan**	**% Vegetarian**	**% Pescatarian**	**% Prefer not to say**
Age	<35	408	345	27.4%	2.94 [2.87, 3.01]	52.6% [47.4%, 57.9%]	25.7% [21.0%, 30.3%]	13.7% [10.1%, 17.3%]	3.1% [1.2%, 4.9%]	10.7% [7.4%, 13.9%]	3.2% [1.3%, 5.1%]	4.8% [2.5%, 7.0%]
	35-49	542	466	24.1%	2.97 [2.91, 3.03]	58.8% [54.3%, 63.2%]	26.0% [22.0%, 30.0%]	8.8% [6.2%, 11.3%]	2.3% [0.9%, 3.6%]	6.5% [4.3%, 8.7%]	3.1% [1.5%, 4.7%]	3.4% [1.7%, 5.0%]
	50-65	680	608	29.0%	3.02 [2.97, 3.08]	56.4% [52.5%, 60.4%]	32.7% [29.0%, 36.4%]	6.5% [4.6%, 8.5%]	1.5% [0.5%, 2.4%]	5.0% [3.3%, 6.8%]	2.0% [0.9%, 3.1%]	2.4% [1.2%, 3.6%]
	>65	486	461	19.6%	3.05 [3.00, 3.10]	54.9% [50.3%, 59.4%]	33.3% [29.0%, 37.6%]	4.9% [2.9%, 6.9%]	0.8% [0.0%, 1.7%]	4.0% [2.2%, 5.8%]	3.3% [1.7%, 5.0%]	3.6% [1.9%, 5.3%]
Gender	Female	1086	926	51.6%	3.08 [3.05, 3.12]	51.4% [48.2%, 54.6%]	31.8% [28.8%, 34.8%]	10.1% [8.2%, 12.1%]	2.2% [1.2%, 3.1%]	8.0% [6.2%, 9.7%]	3.1% [1.9%, 4.2%]	3.7% [2.4%, 4.9%]
	Male	1030	851	48.4%	2.90 [2.85, 2.95]	60.2% [56.9%, 63.5%]	26.6% [23.7%, 29.6%]	7.2% [5.4%, 8.9%]	1.8% [0.9%, 2.6%]	5.4% [3.9%, 7.0%]	2.6% [1.6%, 3.7%]	3.4% [2.2%, 4.6%]
Region	3 major cities	257	208	12.8%	2.99 [2.90, 3.08]	47.1% [40.4%, 53.9%]	26.9% [20.8%, 32.9%]	11.4% [7.0%, 15.7%]	2.7% [0.5%, 4.9%]	8.7% [4.8%, 12.5%]	5.8% [2.6%, 8.9%]	8.9% [5.0%, 12.7%]
	East	443	372	20.6%	3.05 [2.98, 3.11]	54.1% [49.0%, 59.1%]	30.2% [25.5%, 34.9%]	10.1% [7.1%, 13.2%]	1.8% [0.4%, 3.1%]	8.4% [5.6%, 11.2%]	2.5% [0.9%, 4.1%]	3.1% [1.3%, 4.8%]

	North	201	169	10.0%	2.99 [2.89, 3.08]	58.9% [51.5%, 66.4%]	32.9% [25.8%, 40.0%]	5.1% [1.8%, 8.4%]	0.4% [0.0%, 1.3%]	4.7% [1.5%, 8.0%]	1.5% [0.0%, 3.4%]	1.5% [0.0%, 3.4%]
	South	506	436	23.4%	2.95 [2.89, 3.02]	59.3% [54.6%, 63.9%]	26.9% [22.7%, 31.0%]	8.8% [6.1%, 11.4%]	2.5% [1.1%, 4.0%]	6.2% [4.0%, 8.5%]	2.7% [1.2%, 4.2%]	2.4% [1.0%, 3.8%]
	Suburban municipalities	97	81	4.3%	3.10 [3.00, 3.21]	52.8% [42.0%, 63.7%]	30.1% [20.1%, 40.1%]	12.8% [5.5%, 20.0%]	2.7% [0.0%, 6.2%]	10.1% [3.5%, 16.7%]	1.8% [0.0%, 4.7%]	2.5% [0.0%, 5.9%]
	West	612	515	28.9%	2.98 [2.93, 3.04]	56.9% [52.6%, 61.2%]	30.3% [26.3%, 34.2%]	7.1% [4.9%, 9.3%]	1.8% [0.7%, 3.0%]	5.3% [3.4%, 7.2%]	2.6% [1.2%, 3.9%]	3.2% [1.7%, 4.7%]
Ethnic background	European	1760	1480	82.4%	2.97 [2.94, 3.01]	57.2% [54.7%, 59.8%]	29.2% [26.9%, 31.5%]	7.8% [6.5%, 9.2%]	1.5% [0.9%, 2.2%]	6.3% [5.1%, 7.5%]	2.5% [1.7%, 3.3%]	3.3% [2.4%, 4.2%]
	Non-European	351	293	17.4%	3.10 [3.03, 3.17]	48.1% [42.4%, 53.9%]	29.7% [24.5%, 35.0%]	12.9% [9.1%, 16.8%]	4.1% [1.8%, 6.4%]	8.8% [5.6%, 12.1%]	4.7% [2.3%, 7.1%]	4.5% [2.1%, 6.9%]
Education	High	887	821	33.0%	3.07 [3.03, 3.12]	45.2% [41.8%, 48.6%]	39.5% [36.1%, 42.8%]	11.4% [9.2%, 13.5%]	1.9% [0.9%, 2.8%]	9.5% [7.5%, 11.5%]	2.4% [1.4%, 3.5%]	1.5% [0.7%, 2.3%]
	Medium	952	808	48.0%	2.98 [2.93, 3.02]	58.8% [55.4%, 62.2%]	26.6% [23.6%, 29.7%]	7.4% [5.6%, 9.2%]	1.6% [0.7%, 2.5%]	5.8% [4.1%, 7.4%]	3.1% [1.9%, 4.3%]	4.1% [2.7%, 5.4%]
	Low	277	248	18.9%	2.90 [2.82, 2.97]	65.8% [59.9%, 71.7%]	18.3% [13.4%, 23.1%]	7.5% [4.2%, 10.8%]	3.1% [0.9%, 5.3%]	4.4% [1.8%, 6.9%]	2.9% [0.8%, 5.0%]	5.6% [2.7%, 8.5%]
Rural background	No	1449	1214	68.3%	3.03 [2.99, 3.06]	56.4% [53.6%, 59.1%]	28.4% [25.9%, 31.0%]	8.5% [6.9%, 10.1%]	1.6% [0.9%, 2.3%]	6.9% [5.5%, 8.4%]	2.7% [1.8%, 3.6%]	4.0% [2.9%, 5.2%]
	Yes	667	563	31.7%	2.93 [2.88, 2.99]	54.1% [50.0%, 58.2%]	31.2% [27.3%, 35.0%]	9.2% [6.8%, 11.5%]	2.8% [1.5%, 4.2%]	6.3% [4.3%, 8.3%]	3.2% [1.7%, 4.7%]	2.4% [1.1%, 3.6%]
Visited a livestock farm	No	1609	1357	76.0%	3.03 [2.99, 3.06]	56.6% [53.9%, 59.2%]	27.8% [25.4%, 30.2%]	8.9% [7.4%, 10.4%]	1.8% [1.1%, 2.5%]	7.1% [5.8%, 8.5%]	2.5% [1.6%, 3.3%]	4.3% [3.2%, 5.3%]
	Yes	507	420	24.0%	2.90 [2.84, 2.96]	52.7% [47.9%, 57.5%]	34.0% [29.4%, 38.5%]	8.1% [5.5%, 10.7%]	2.6% [1.1%, 4.2%]	5.5% [3.3%, 7.6%]	4.0% [2.2%, 5.9%]	1.2% [0.2%, 2.2%]

Ever worked with livestock	No	2000	1683	93.8%	3.00 [2.97, 3.03]	56.4% [54.0%, 58.8%]	29.6% [27.4%, 31.7%]	8.1% [6.8%, 9.4%]	1.8% [1.1%, 2.4%]	6.4% [5.2%, 7.6%]	2.3% [1.6%, 3.0%]	3.6% [2.7%, 4.5%]
	Yes	116	96	6.2%	2.94 [2.81, 3.07]	44.0% [34.1%, 54.0%]	25.2% [16.5%, 33.9%]	17.3% [9.7%, 24.9%]	5.4% [0.8%, 9.9%]	12.0% [5.5%, 18.5%]	11.5% [5.1%, 17.8%]	2.0% [0.0%, 4.7%]
Regular contact with animals	no	841	712	39.2%	2.88 [2.83, 2.93]	59.1% [55.5%, 62.7%]	26.6% [23.3%, 29.8%]	7.2% [5.3%, 9.1%]	1.4% [0.5%, 2.2%]	5.8% [4.1%, 7.5%]	2.4% [1.3%, 3.6%]	4.7% [3.1%, 6.2%]
	yes	1275	1065	60.8%	3.07 [3.03, 3.10]	53.4% [50.4%, 56.4%]	31.0% [28.3%, 33.8%]	9.7% [7.9%, 11.5%]	2.4% [1.5%, 3.3%]	7.3% [5.8%, 8.9%]	3.1% [2.1%, 4.2%]	2.8% [1.8%, 3.8%]
Regular contact with pets	no	911	766	43.1%	2.89 [2.84, 2.94]	56.9% [53.4%, 60.4%]	26.2% [23.1%, 29.3%]	8.6% [6.7%, 10.6%]	1.9% [0.9%, 2.9%]	6.7% [5.0%, 8.5%]	3.2% [1.9%, 4.4%]	5.1% [3.5%, 6.7%]
	yes	1205	1011	56.9%	3.07 [3.03, 3.11]	54.7% [51.6%, 57.8%]	31.6% [28.8%, 34.5%]	8.8% [7.0%, 10.5%]	2.0% [1.2%, 2.9%]	6.7% [5.2%, 8.3%]	2.6% [1.6%, 3.6%]	2.3% [1.4%, 3.2%]
Regular contact with livestock	no	1985	1673	93.1%	3.01 [2.98, 3.04]	56.0% [53.6%, 58.4%]	29.7% [27.6%, 31.9%]	8.2% [6.9%, 9.5%]	1.9% [1.3%, 2.6%]	6.3% [5.2%, 7.5%]	2.7% [1.9%, 3.4%]	3.4% [2.5%, 4.2%]
	yes	131	107	6.9%	2.84 [2.72, 2.97]	50.6% [41.1%, 60.1%]	23.1% [15.1%, 31.1%]	15.2% [8.4%, 22.0%]	2.9% [0.0%, 6.0%]	12.3% [6.1%, 18.5%]	5.5% [1.2%, 9.8%]	5.7% [1.3%, 10.1%]
Regular contact with other animals	no	1976	1660	93.3%	2.99 [2.96, 3.02]	56.3% [53.9%, 58.7%]	29.2% [27.0%, 31.4%]	8.3% [7.0%, 9.7%]	2.0% [1.3%, 2.6%]	6.4% [5.2%, 7.6%]	2.7% [1.9%, 3.5%]	3.4% [2.5%, 4.3%]
	yes	140	116	6.7%	3.07 [2.95, 3.19]	46.2% [37.2%, 55.3%]	30.6% [22.2%, 38.9%]	13.7% [7.4%, 19.9%]	2.3% [0.0%, 5.0%]	11.4% [5.6%, 17.2%]	4.6% [0.8%, 8.4%]	4.9% [1.0%, 8.8%]
Religion	Atheist (not religious)	861	723	39.1%	3.10 [3.06, 3.15]	51.4% [47.8%, 55.1%]	35.6% [32.1%, 39.1%]	9.4% [7.3%, 11.6%]	2.2% [1.1%, 3.2%]	7.3% [5.4%, 9.2%]	2.9% [1.6%, 4.1%]	0.7% [0.1%, 1.2%]
	Christian	737	636	33.7%	2.87 [2.83, 2.92]	64.0% [60.2%, 67.7%]	28.1% [24.6%, 31.5%]	4.3% [2.8%, 5.9%]	1.1% [0.3%, 1.9%]	3.3% [1.9%, 4.7%]	1.8% [0.8%, 2.9%]	1.8% [0.8%, 2.8%]

	I prefer not to say	251	208	12.7%	2.88 [2.79, 2.97]	53.8% [47.0%, 60.5%]	18.4% [13.1%, 23.6%]	8.0% [4.3%, 11.7%]	0.6% [0.0%, 1.6%]	7.4% [3.9%, 11.0%]	2.9% [0.6%, 5.2%]	16.9% [11.8%, 22.0%]
	Other	267	220	14.5%	3.07 [2.99, 3.15]	49.3% [42.7%, 56.0%]	24.6% [18.9%, 30.3%]	17.5% [12.5%, 22.5%]	4.8% [1.9%, 7.6%]	12.7% [8.3%, 17.1%]	5.1% [2.2%, 8.0%]	3.5% [1.1%, 5.9%]
Voting	Left	597	504	26.4%	3.35 [3.31, 3.40]	34.8% [30.7%, 39.0%]	44.4% [40.1%, 48.8%]	15.3% [12.1%, 18.4%]	3.8% [2.1%, 5.5%]	11.5% [8.7%, 14.3%]	5.0% [3.1%, 6.9%]	0.4% [0.0%, 1.0%]
	Center	350	294	15.9%	2.93 [2.87, 3.00]	52.2% [46.5%, 57.9%]	35.2% [29.7%, 40.7%]	6.6% [3.8%, 9.5%]	1.3% [0.0%, 2.6%]	5.3% [2.8%, 7.9%]	3.0% [1.0%, 4.9%]	3.0% [1.0%, 4.9%]
	Right	717	605	34.9%	2.78 [2.73, 2.83]	69.4% [65.8%, 73.1%]	20.6% [17.4%, 23.8%]	6.7% [4.7%, 8.7%]	1.5% [0.6%, 2.5%]	5.2% [3.4%, 6.9%]	1.4% [0.5%, 2.3%]	1.8% [0.8%, 2.9%]
	Unknown	452	378	22.8%	2.93 [2.87, 2.99]	61.0% [56.1%, 65.9%]	20.9% [16.8%, 25.0%]	5.6% [3.3%, 7.9%]	1.0% [0.0%, 2.0%]	4.6% [2.5%, 6.7%]	2.5% [0.9%, 4.1%]	10.1% [7.0%, 13.1%]
Household size	1 person	472	401	22.2%	3.07 [3.01, 3.14]	49.9% [45.0%, 54.8%]	33.3% [28.6%, 37.9%]	10.4% [7.4%, 13.4%]	2.1% [0.7%, 3.4%]	8.4% [5.6%, 11.1%]	2.3% [0.9%, 3.8%]	4.1% [2.2%, 6.0%]
	2 persons	836	728	36.9%	3.04 [2.99, 3.08]	54.5% [50.9%, 58.1%]	31.8% [28.4%, 35.2%]	7.6% [5.6%, 9.5%]	1.5% [0.7%, 2.4%]	6.0% [4.3%, 7.7%]	3.3% [2.0%, 4.6%]	2.9% [1.7%, 4.1%]
	3 or more persons	807	660	40.8%	2.91 [2.86, 2.96]	59.8% [56.0%, 63.5%]	24.9% [21.6%, 28.2%]	8.8% [6.7%, 11.0%]	2.3% [1.2%, 3.5%]	6.5% [4.6%, 8.4%]	2.7% [1.5%, 4.0%]	3.8% [2.3%, 5.2%]
Income	Above average	721	636	29.6%	3.01 [2.96, 3.07]	52.8% [48.9%, 56.6%]	34.3% [30.7%, 38.0%]	8.9% [6.7%, 11.1%]	1.8% [0.8%, 2.9%]	7.0% [5.1%, 9.0%]	2.6% [1.4%, 3.9%]	1.4% [0.5%, 2.3%]
	Average	273	228	13.1%	2.94 [2.85, 3.03]	60.6% [54.3%, 67.0%]	26.4% [20.7%, 32.1%]	8.8% [5.1%, 12.5%]	1.7% [0.0%, 3.4%]	7.1% [3.7%, 10.4%]	1.6% [0.0%, 3.3%]	2.5% [0.5%, 4.6%]
	Below average	598	501	31.5%	3.02 [2.96, 3.07]	56.2% [51.9%, 60.6%]	28.1% [24.2%, 32.0%]	8.9% [6.4%, 11.4%]	2.3% [1.0%, 3.6%]	6.6% [4.4%, 8.7%]	3.8% [2.2%, 5.5%]	2.9% [1.5%, 4.4%]
	Unknown	524	438	25.8%	2.97 [2.92, 3.03]	55.7% [51.0%, 60.4%]	26.4% [22.3%, 30.5%]	8.3% [5.7%, 10.8%]	1.9% [0.6%, 3.1%]	6.4% [4.1%, 8.7%]	2.5% [1.1%, 4.0%]	7.1% [4.7%, 9.6%]

Intern gebruik

Parental status	No parent	773	626	41.3%	3.03 [2.98, 3.09]	53.3% [49.4%, 57.2%]	29.1% [25.6%, 32.7%]	10.1% [7.7%, 12.4%]	2.7% [1.4%, 3.9%]	7.4% [5.4%, 9.5%]	3.5% [2.1%, 4.9%]	4.0% [2.5%, 5.6%]
	Parent	1331	1176	58.3%	2.97 [2.93, 3.01]	57.3% [54.5%, 60.1%]	29.5% [26.9%, 32.1%]	7.7% [6.1%, 9.2%]	1.5% [0.8%, 2.1%]	6.2% [4.8%, 7.6%]	2.4% [1.5%, 3.3%]	3.1% [2.1%, 4.1%]
	Unknown	12	12	0.4%	2.78 [2.48, 3.08]	58.7% [30.8%, 86.7%]	15.5% [0.0%, 36.0%]	18.1% [0.0%, 39.9%]	7.7% [0.0%, 22.9%]	10.3% [0.0%, 27.6%]	0.0% [0.0%, 0.0%]	7.7% [0.0%, 22.9%]
FAW concern	Not concerned at all	67	55	3.7%		86.1% [76.9%, 95.2%]	5.3% [0.0%, 11.3%]	2.8% [0.0%, 7.1%]	0.0% [0.0%, 0.0%]	2.8% [0.0%, 7.1%]	2.7% [0.0%, 7.0%]	3.1% [0.0%, 7.7%]
	Low concern	308	258	14.5%		76.5% [71.4%, 81.7%]	16.3% [11.8%, 20.8%]	1.9% [0.3%, 3.6%]	0.0% [0.0%, 0.0%]	1.9% [0.3%, 3.6%]	1.9% [0.2%, 3.6%]	3.4% [1.2%, 5.6%]
	Moderate concern	924	777	43.9%		62.3% [58.9%, 65.7%]	27.6% [24.5%, 30.8%]	4.6% [3.1%, 6.1%]	1.6% [0.7%, 2.4%]	3.1% [1.8%, 4.3%]	1.9% [1.0%, 2.9%]	3.6% [2.3%, 4.9%]
	Strong concern	708	602	31.9%		31.5% [27.8%, 35.2%]	43.4% [39.4%, 47.3%]	19.1% [16.0%, 22.3%]	3.9% [2.4%, 5.4%]	15.2% [12.3%, 18.1%]	5.1% [3.3%, 6.9%]	0.9% [0.1%, 1.6%]

Note: all results are weighted except the column n.
